# The association of health-related factors with quality of life among the elderly population in the Jaffna district of Sri Lanka

**DOI:** 10.1186/s12889-021-10507-3

**Published:** 2021-03-06

**Authors:** Sathees Santhalingam, Sivayogan Sivagurunathan, Shamini Prathapan, Sivapalan Kanagasabai, Luxmi Kamalarupan

**Affiliations:** 1 Department of Nursing, Faculty of Allied Health Sciences, University of Jaffna, Jaffna, Sri Lanka; 2 Department of Community Medicine, Faculty of Medical Sciences, University of Sri Jayewardenepura, Nugegoda, Sri Lanka; 3 Department of Physiology, Faculty of Medicine, University of Jaffna, Jaffna, Sri Lanka

**Keywords:** Quality of life, Health, Elderly, Sri Lanka

## Abstract

**Background:**

The proportion of elderly individuals is increasing globally. They should be well cared for to enable them to enjoy their full lifespans. Good health is a vital component of one’s overall quality of life. Our study aimed to assess the association of health-related factors with quality of life among elderly individuals in the Jaffna District of Sri Lanka.

**Methods:**

We conducted a community-based, cross-sectional study among 813 elderly individuals in the Jaffna district of Sri Lanka. Sociodemographic factors and the patterns of health conditions were recorded through an interviewer-administered questionnaire. Quality of life was measured through the World Health Organisation Quality of Life-Bref (WHOQOL-Bref) questionnaire.

**Results:**

There were slightly more male respondents (53.5%) than females in the study. The median age of the participants was 70 (11) years. Approximately one-third of them had at least one chronic health condition. Musculoskeletal complaints were found to be the most common health condition, followed by diabetes, hypertension, vision problems, and asthma. Among the respondents, 20.1% were attending regular follow-up visits in a clinic, and 24% of them were meeting a doctor at least monthly. Among them, 6.8% had at least one limitation in their activities of daily living. However, the majority (58.6%) reported that they were satisfied with their health status. The following factors were found to be significantly associated with worse quality of life: the presence of health conditions, the presence of musculoskeletal conditions, hearing impairment, vision impairment, bronchial asthma, limitations in activities of daily living, and the use of addictive substances. Satisfaction with health, regular follow-up visits in a clinic, meeting a doctor at least monthly, and having diabetes were significantly associated with better quality of life.

**Conclusion:**

Minimising the limitations of daily living, abstaining from using addictive substances, preventing diseases, and improving access to health services may enhance the quality of life of elderly individuals. Furthermore, these factors should be considered by policy makers seeking to improve the quality of life of elderly individuals.

## Background

The life expectancy of humans has been increasing worldwide [1] due to the following factors: (a) the rapid advancement of health services, leading to improved standards of living; (b) healthy dietary practices; (c) increased levels of physical activity; and (d) the cessation of smoking and alcohol consumption [2, 3]. At the same time, the global fertility rate has decreased. This has led to an increase in the global elderly population [1, 4, 5]. In most countries, a person who is over 60 years of age is considered to be elderly [6].

Currently, two-thirds of the elderly population worldwide live in developing countries [7]. The United Nations predicts that eight out of 10 elderly individuals will live in developing countries by 2050 [8]. Among the different Southeast Asian countries, Sri Lanka currently has a relatively high proportion of elderly individuals and is predicted to continue to have a relatively high proportion [9]. The proportion of elderly individuals was reported to be 1 in 10 in 2000, and this proportion increased to 1 in 5 by 2010 [10]. Additionally, the median age of the Sri Lankan population is expected to increase from 26.9 to 39.2 years between 2000 and 2030 [10]. The rapidity of population ageing has been attributed to the large demographic transition in the country [11, 12]. The growing elderly population is gradually replacing the younger population; therefore, the dependency ratio is expected to increase in the future [12]. As a developing country, it is clear that this could lead to significant challenges in various societal aspects [11].

The phenomenon of population ageing and the associated demographic transitions occurred concurrently with slow economic growth in Sri Lanka. This is in contrast to how the demographic shift in more developed countries has occurred during periods of rapid economic growth. Thus, Sri Lanka faces unique challenges when compared with countries in the developed world [9]. Population ageing and social transformation, as well as changes in social values, are new and growing challenges facing the Sri Lankan elderly population. Therefore, there is growing concern about this population.

Elderly individuals are at elevated risk for physical and psychological problems. The health conditions that most commonly affect elderly individuals are musculoskeletal conditions, respiratory diseases, gastrointestinal conditions, and cardiovascular conditions [13]. In Sri Lanka, common health conditions in the elderly population include hypertension, diabetes, visual and hearing impairments, arthritis, and asthma [14]. Apart from these physical problems, many elderly individuals also suffer from various psychological problems. If these health conditions are not managed well, they can affect an older person’s wellbeing.

It is known that ageing and its associated changes usually negatively affect the health status of elderly individuals [15]. The following health-related factors have been found to be closely associated with worse quality of life in various past studies: (a) morbidities, (b) pain, (c) negative perceptions of medication, and (d) negative perceptions of health. In contrast, the following factors have been found to be associated with a better quality of life: (a) health service availability, (b) the absence of chronic diseases, (c) satisfaction with health, and (d) the absence of limitations of activities of daily living (ADL) [14, 16, 17].

In Sri Lanka, changes have occurred in the socio-economic and family systems, from the extended family to the nuclear family. Therefore, many of those elderly individuals who, in the past, would have depended on their family members for care now suffer from a lack of caregivers [18]. This tends to impair the quality of care provided to elderly individuals in Sri Lanka and, in turn, leads to a variety of social, psychological, and physical problems.

It is vital to maintain the health of the elderly population not only for their own sakes but also for the wellbeing of the society as a whole. Moreover, it is clear that the availability of human and social resources positively influences the health of the elderly population [19]. It is essential to note that the currently accepted concepts of healthy elderhood and healthy ageing include not only the absence of disease but also an overall improvement in their quality of life (QOL) [19].

Quality of life is a good indicator of the health and wellbeing of the elderly population. It is a complex, broad-ranging concept that can include a person’s physical health, psychological state, personal beliefs, social relationships, and relationship to salient environmental features [20].

Very few studies have been conducted on the quality of life of elderly individuals in Sri Lanka. One of the few researchers on the subject has reported that the QOL of the elderly in Sri Lanka was moderate and that it was significantly associated with health-related factors [18].

Jaffna District is one of the 25 administrative districts of Sri Lanka. It is in the Northern Province; it is the northernmost district of Sri Lanka, and the total population is 618,209 [21]. The majority of the population is Sri Lankan Tamils (98.9%), followed by Muslims and Sinhalese. Agriculture is the main economy in the Jaffna district. According to the Sri Lankan Government Information Centre, a person who has surpassed 60 years of age is considered elderly in Sri Lanka [22]. The proportion of elderly individuals in Jaffna District is higher (14.1%) [23] than that in Sri Lanka as a whole (12.4%) [24].

In Jaffna, three decades of war, which ended in 2009, resulted in the rapid migration and death of younger people and increased the percentage of elderly people; this also led to a lack of caregivers [25]. Jaffna District ranks fourth among the districts of Sri Lanka with regard to the proportion of the population that is elderly. There is a relatively greater need to study the wellbeing of the elderly population in Jaffna District in the post-war period. The objective of this study was to increase the understanding of health-related factors and their influence on the quality of life of elderly residents in Jaffna District through a descriptive, cross-sectional study. It is hoped that the results of this study will facilitate the development of positive interventions to improve and maintain the desired standards for quality of life in the Jaffna District, as well as throughout Sri Lanka.

## Methods

### Study sample and procedures

This cross-sectional descriptive study included persons who were residents in Jaffna District and older than 60 years. Institutionalized elderly individuals, who represent only 0.45% of the elderly population in Jaffna [21], were excluded from this study, as their living arrangements and patterns of care are different from those of noninstitutionalized elderly individuals.

The prevalence of poor self-reported QOL was found to be 48% in a previous study [18]. Assuming a prevalence of 48%, a precision of 0.05, and a confidence level of 95%, the sample size needed was calculated to be 384 based on the Daniel formula [26]. It was doubled to overcome the effect of the design and further increased to approximately 880 to account for a nonresponse rate of 15%.

Two-stage random sampling (geographical cluster sampling technique) was applied. All Grama Niladari (GN) areas (smallest administrative units of Sri Lanka) (440) in Jaffna District were used as the sampling framework for the first stage, while elderly individuals older than 60 years in each GN area were used as the sampling framework for the second stage.

Ethics approval was obtained from the Ethics Review Committee of the Faculty of Medicine of the University of Jaffna of Sri Lanka (Ref. No-J/ERC/18/92/DR/0059).

### Measurements

An interviewer-administered questionnaire was used to assess the sociodemographic characteristics and health-related factors. Further series of questions related to their health history and exposure to addictive substances were included. Smoking, betel chewing, and alcohol consumption were considered the use of addictive substances.

The previously validated WHOQOL-Bref questionnaire (Tamil version) was used to measure the quality of life of the elderly participants. This questionnaire evaluates the perceived QOL through 26 questions. Responses are given on a 5-point Likert scale. Questions were categorised to measure QOL in four broad domains: the physical domain, psychological domain, social domain, and environmental domain. A higher total score indicates a better QOL in all four domains in elderly individuals [27].

Data were collected during face-to-face interviews at the participants’ homes. The presence or absence of the following health conditions was recorded: diabetes mellitus, hypertension, hyperlipidaemia, heart disease, musculoskeletal conditions, kidney diseases, vision impairment, hearing impairment, bronchial asthma, and any other diseases. Moreover, follow-up visits at a clinic and the frequency with which they visited a doctor were also recorded by the interviewers during the face-to-face interviews. Recall bias was considered minimal, as the information gathered was highly personal. Participants who found it difficult to recall the date of their last clinic or doctor visit were assisted in recalling it in relation to the timing of annual festivals. Individual scores for the domains of quality of life were measured. The transformed scores were calculated using the transformed score table; overall quality of life was determined based on the average of the domain scores.

### Statistical analysis

All the data collected were analysed with the Statistical Package for Social Sciences (SPSS) for Windows, version 16. Both descriptive and inferential statistics were used to report the results for the sociodemographic variables, self-reported health conditions, and quality of life. The results for categorical variables are expressed as proportions and frequencies. Continuous variables are reported as the means and standard deviations.

One-way ANOVA and independent *t-*tests were used to identify the mean differences in quality of life with respect to the presence or absence of the health conditions considered in this study. Variables with a *p*-value less than 0.05 in univariate analysis were included in the multiple linear regression model, and further multicollinearity of variables was tested. This model was used to determine the independent association of the health-related factors with quality of life, and a *p-*value < 0.05 was considered statistically significant.

## Results

A total of 813 prospective participants consented to take part in the study, with a response rate of 92.4%. The median age of the participants was 70 (11) years. The majority of the participants were the age group from 60 to 69 years. There was a slightly higher proportion of males (53.5%) than females. The majority of the participants were Hindu (85.9%). Approximately two-thirds of them (61.6%) lived with their spouse, and a small proportion (1.7%) of them had not gone to school. Approximately half of them had never worked (47%), 36.3% were currently working, and 16.7% were retired from previous employment. Concerning monthly income, 54.9% had a monthly income below the national poverty line (food and non-food expenditures per person per month) (see Table 1).

**Table 1:** Sociodemographic characteristics of the participants

Sociodemographic yactors (*n* = 813)		n	%
Age (Years)	60–69	382	47.0
	70–79	308	37.9
	80 and above	123	15.1
Sex	Male	435	53.5
	Female	378	46.5
Religion	Hindu	685	85.9
	Christian	107	13.5
	Islam	05	0.6
Marital status	Unmarried	20	2.5
	Married	500	61.6
	Widowed	261	32.2
	Divorced/separated	30	3.7
Education	No schooling	14	1.7
	Primary	98	12.1
	Secondary	607	74.7
	Collegial	58	7.1
	Tertiary	36	4.4
Occupation	Retired	136	16.7
	Currently working	295	36.3
	Never worked	382	47.0
Monthly income	Below national poverty line	384	54.9
	Above national poverty line	315	45.1

Table 2 shows the quality of life scores. The overall (average) score for quality of life was 11.5 ± 2.2 out of 20. Regarding the different quality of life domain scores, the environmental domain was found to have the highest score (12.1 ± 2.1), followed by the psychological domain (12.0 ± 2.8), the physical domain (11.8 ± 2.3), and the social domain (10.1 ± 3.0).

**Table 2:** Scores for QOL domains (*n =* 813)

QOL scores	Minimum	Maximum	Mean	SD
Physical	5.00	19.00	11.79	2.30
Psychological	5.00	19.00	11.97	2.77
Social Relationship	4.00	20.00	10.14	3.04
Environment	4.00	19.00	12.10	2.11
Overall (average) score	5.50	18.75	11.50	2.17

Concerning the health status of the participants, 62.6% of them reported that they had at least one health condition. The most prevailing type of health condition was musculoskeletal disorders (22.3%). This was followed by diabetes mellitus (20.7%). The next most common conditions were hypertension (18.8%), vision impairment (16.2%), and asthma (9.8%). The combination of diabetes mellitus and hypertension was the most common, as 86 elderly individuals had both diseases.

Among the respondents, 169 (20.9%) were attending regular follow-up in a clinic, while 24.9% were visiting their doctor at least once a month.

The mean differences in the quality of life domain scores of the elderly individuals with various conditions are shown in Table 3. The overall quality of life score was the lowest among elderly individuals with kidney disease, followed by those with musculoskeletal conditions, hearing impairment, and visual impairment. Furthermore, overall QOL was associated with the presence of diabetes mellitus, musculoskeletal conditions, vision impairment, hearing impairment, and bronchial asthma. An independent t-test revealed that the mean differences were statistically significant.

**Table 3:** Health conditions and their associations with the QOL of participants

Diseases (*n =* 813)	n	%	Physical domain Mean (±SD)	Psychological domain Mean(±SD)	Social relationship domain Mean(±SD)	Environmental domain Mean(±SD)	Overall QOL Mean ± SD)
Diabetic mellitus							
Yes	168	20.7	12.1 (2.3)	12.8 (3.0)	10.7 (3.3)	12.9 (2.1)	12.1 (2.3)
No	645	79.3	11.7 (2.3)	11.8 (2.6)	10.0 (2.9)	11.9 (2.0)	11.3 (2.1)
*p-value*			< 0.001	0.048	*p* < 0.001	0.011	*p <* 0.001
Hypertension							
Yes	153	18.8	11.8 (2.3)	12.4 (3.0)	10.2 (3.2)	12.4 (2.3)	11.7 (2.3)
No	660	81.2	11.8 (2.3)	11.9 (2.7)	10.1 (3.0)	12.0 (2.0)	11.4 (2.1)
*p-value*			0.871	0.053	0.894	0.043	0.220
Hyperlipidaemia							
Yes	29	3.6	11.6 (2.2)	12.7 (2.8)	9.0 (3.8)	13.3 (2.3)	11.7 (2.2)
No	784	96.4	11.8 (2.3)	12.0 (2.8)	10.2 (3.0)	12.0 (2.1)	11.5 (2.2)
*p-value*			0.940	0.148	0.102	0.10	0.661
Heart disease							
Yes	18	2.2	11.1 (2.3)	12.8 (1.7)	8.9 (3.3)	12.4 (1.3)	11.3 (1.7)
No	795	97.8	11.8 (2.2)	11.9 (2.8)	10.2 (3.0)	12.1 (2.2)	11.5 (2.2)
*p-value*			0.171	0.182	0.078	0.481	0.701
Musculoskeletal conditions							
Yes	181	22.3	9.9 (1.6)	10.6 (2.6)	8.9 (2.5)	11.2 (2.0)	10.1 (1.8)
No	632	77.7	12.3 (2.1)	12.4 (2.7)	10.5 (3.1)	12.4 (2.0)	11.9 (2.1)
*p-value*			*p <* 0.001	*p <* 0.001	*p <* 0.001	*p <* 0.001	*p <* 0.001
Kidney disease							
Yes	7	0.9	10.3 (1.8)	9.4 (2.6)	8.9 (3.1)	10.6 (3.1)	9.8 (2.4)
No	806	99.1	11.8 (2.3)	12.0 (2.8)	10.1 (3.0)	12.1 (2.1)	11.5 (2.2)
*p-value*			0.082	0.015	0.263	0.054	0.056
Vision problems							
Yes	132	16.2	10.6 (2.2)	11.5 (2.7)	9.2 (3.1)	12.0 (2.1)	10.8 (2.0)
No	681	83.8	12.0 (2.2)	12.1 (2.8)	10.3 (3.0)	12.1 (2.1)	11.6 (2.2)
*p-value*			*P <* 0.001	0.040	*P <* 0.001	0.680	*P <* 0.001
Hearing impairment							
Yes	40	4.9	10.0 (2.3)	10.8 (2.4)	8.2 (2.2)	11.4 (2.1)	10.1 (1.7)
No	773	95.1	11.9 (2.3)	12.0 (2.8)	10.2 (3.0)	12.1 (2.1)	11.6 (2.2)
*p-value*			*P <* 0.001	0.003	*P <* 0.001	0.035	*P <* 0.001
Bronchial asthma							
Yes	80	9.8	11.0 (1.9)	11.8 (2.8)	9.5 (2.6)	11.7 (2.2)	11.0 (2.0)
No	773	90.2	11.9 (2.3)	12.0()2.8	10.2 (3.1)	12.1 (2.1)	11.6 (2.2)
*p-value*			0.001	0.516	0.022	0.089	0.019

Overall quality of life was higher in those who had no diseases, no ADL limitations, no addictive substance use, satisfaction with their health status and regular clinic and doctor follow-up visits. Table 4 shows the mean differences based on the above factors in the individual quality of life domain scores.

**Table 4:** Health-related factors and their associations with the QOL of participants

Associated factors	n	%	Physical domain Mean (±SD)	Psychological domain Mean (±SD)	Social relationship domain Mean (±SD)	Environmental domain Mean (±SD)	Overall QOL Mean (±SD)
Presence of health problem							
Yes	509	62.6	11.1 (2.3)	11.6 (2.8)	9.6 (3.0)	12.0 (2.1)	11.1 (2.2)
No	304	37.4	13.0 (1.8)	12.5 (2.5)	11.0 (2.8)	12.3 (2.0)	12.2 (2.0)
*p-*value			< 0.001	< 0.001	< 0.001	0.036	< 0.001
At least one ADL limitation							
Yes	55	6.8	8.4 (2.5)	9.8 (2.5)	7.2 (2.0)	10.8 (2.0)	9.1 (1.7)
No	758	93.2	12.0 (2.0)	12.1 (2.7)	10.3 (3.0)	12.2 (2.0)	11.7 (2.1)
*p-*value			< 0.001	< 0.001	< 0.001	< 0.001	< 0.001
Satisfaction on health status							
Yes	477	58.8	12.9 (1.8)	12.8 (2.6)	11.0 (3.0)	12.6 (2.0)	12.3 (2.0)
No	334	41.2	10.1 (1.9)	10.8 (2.6)	8.9 (2.7)	11.4 (2.0)	10.3 (1.8)
*p-*value			< 0.001	< 0.001	< 0.001	< 0.001	< 0.001
Clinic following							
Yes	169	20.9	12.0 (2.5)	13.2 (2.9)	10.6 (3.5)	13.2 (2.2)	12.0 (2.3)
No	644	79.1	11.7 (2.2)	11.6 (2.6)	10.0 (2.8)	11.8 (1.9)	11.3 (2.0)
*p-*value			0.143	< 0.001	0.048	< 0.001	< 0.001
Visiting a doctor at least monthly							
Yes	195	24.9	11.7 (2.6)	12.9 (3.0)	10.3 (3.6)	13.1 (2.2)	12.0 (2.4)
No	589	75.1	11.8 (2.1)	11.6 (2.6)	10.1 (2.8)	11.8 (1.9)	11.3 (2.0)
*p-*value			0.002	< 0.001	0.331	< 0.001	< 0.001
Addictive substance use							
yes	348	42.8	11.8 (2.3)	11.6 (2.7)	10.0 (3.2)	11.8 (2.0)	11.3 (2.1)
no	465	57.2	11.8 (2.3)	12.2 (2.8)	10.2 (2.9)	12.3 (2.1)	11.6 (2.1)
*p-*value			0.849	0.001	0.341	0.001	0.033

The details of the clinical follow-up among the elderly patients with various conditions are shown in Table 5. The highest proportion of elderly individuals attending regular follow-up in a clinic had heart disease (83.3%). This was followed by diabetes mellitus (69.6%), hyperlipidaemia (62.1%), and hypertension (54.9%). A similar pattern was observed with regard to those who visited doctors at least once a month. The proportions were 88.9, 70.2, 62.1, and 59%, respectively.

**Table 5:** Proportions of participants with regular clinical follow-up and monthly doctor visits stratified by health conditions

Diseases	Number of elderly individuals with that specific disease	Number of elderly individuals regularly attending a clinic		Number of elderly individuals visiting a doctor at least monthly	
		n	%	n	%
Muscular skeletal conditions	181	33	18.2	41	22.6
Diabetes	168	117	69.6	118	70.2
Hypertension	153	84	54.9	91	59.4
Vision impairment	132	38	28.8	47	35.6
Bronchial asthma	80	23	28.7	22	27.5
Hearing impairment	40	4	10	7	17.5
Hyperlipidaemia	29	18	62.1	18	62.1
Heart disease	18	15	83.3	16	88.9
Kidney disease	7	2	28.5	3	42.8
Other	13	3	23.1	4	30.8

Variables that were found to be associated with overall QOL with a *p*-value less than 0.05 were included in the multiple linear regression to identify whether there were independent associations with the overall QOL of elderly individuals. Clinical follow-up was removed from the regression due to multicollinearity.

Multiple linear regression revealed that the presence of specific health conditions, such as musculoskeletal conditions; ADL limitations; and exposure to addictive substances were independently associated with a reduced overall quality of life among the participants. Furthermore, satisfaction with their health, visiting a doctor at least once a month, and having diabetes mellitus were independently associated with higher overall QOL (see Table 6).

**Table 6:** Contributing factors to the QOL of participants

Factors	B-	S.E	Beta	t	*P-*Value	95% CI B	
						Lower	Upper
(Constant)	10.977	.215		50.943	.000	10.554	11.400
Presence of diabetic mellitus	.469	.198	.090	2.368	.018	.080	.857
Presence of musculoskeletal conditions	−.535	.192	−.105	−2.779	.006	−.913	−.157
Presence of vision impairment	−.040	.182	−.007	−.222	.824	−.397	.316
Presence of hearing impairment	−.431	.305	−.043	−1.412	.158	−1.030	.168
Presence of bronchial asthma	.114	.223	.016	.511	.609	−.324	.551
Presence of specific disease	−.416	.202	−.093	−2.058	.040	−.813	−.019
Having at least one ADL limitation	−2.012	.272	−.230	−7.386	.000	−2.546	−1.477
Satisfied with health status	1.446	.184	.334	7.859	.000	1.085	1.807
Visiting the doctor at least monthly	1.035	.187	.209	5.521	.000	.667	1.403
Exposure to addictive substances	−.401	.129	−.093	−3.115	.002	−.654	−.148

(Multiple linear regression *R* = 0.585, *R*^2^ = 0.342; Adjusted *R*^2^ = 0.344, SE = 1.747) Significance *P* < 0.05

(Higher score indicates better QOL)

## Discussion

The current study revealed that the overall quality of life score among elderly individuals was 11.5 ± 2.2 out of 20. Furthermore, the mean scores for all four individual domains seem to be low when compared to the WHOQOL-Bref global mean scores for the elderly age group [27]. This result is comparable with similar studies previously conducted in Sri Lanka [14, 16].

The QOL of the elderly population was found to be associated with health-related factors such as the presence of a specific health condition, limitations in activities of daily living, exposure to addictive substances, perceived satisfaction with their health and the frequency of visiting a doctor.

The prevalence of chronic illness in the study population was similar to the national prevalence [28].

The results show that the following health conditions were associated with the overall quality of life among the elderly population in the Jaffna district: diabetes mellitus, musculoskeletal conditions, vision problems, hearing impairment, and bronchial asthma. These conditions were found to be associated with the QOL of elderly individuals in previous studies [14, 17].

Among these disease conditions, musculoskeletal conditions were the most prevalent complaint among the elderly population in Jaffna. This is in contrast to the finding from previous studies that hypertension is the health condition with the highest prevalence among the elderly population in Sri Lanka [18, 28]. This difference in disease prevalence is notable, as musculoskeletal conditions are associated with the dependency of the elderly; thus, they could be strongly associated with their QOL.

The prevalence of diabetes mellitus among the elderly population in Jaffna was high. Moreover, the prevalence of hypertension was considerably lower than the national prevalence. This difference may be associated with the differences in lifestyle practices and food preferences of the people in Jaffna compared to those of people living in other parts of the country.

The combination of multiple diseases is common among the elderly population [29]. Hypertension was previously found to be most commonly comorbid with diabetes mellitus [30]. This finding was supported by the current study, which showed that 86 elderly participants had both diabetes mellitus and hypertension and that this was the most common combination. Therefore, approximately one in ten elderly individuals in the Jaffna district have both diseases.

Surprisingly, having diabetes contributed to a higher quality of life. It was reported that the complications of chronic diseases are the main reason for the reduced quality of life among the elderly population. Hence, the absence of complications, active clinical follow-up, and regular monitoring could be possible reasons for this result. Notably, the Jaffna Teaching Hospital runs a Diabetic Centre where most of the diagnosed diabetic patients were registered. They receive regular follow-up and annual screening for associated long-term complications, and good health education is provided. This seems to be effective with regard to the management of complications associated with diabetes mellitus.

Elderly individuals with kidney disease reported the lowest overall quality of life compared to those with other diseases. A previous study conducted among elderly individuals in the Anuradhapura District also reported the same finding [14]. However, the contribution of the presence of kidney disease to the reduction in the quality of life of elderly individuals in Jaffna was not statistically significant. The prevalence of kidney disease is much higher in Anuradhapura District (15–23%) [31], while it is very low in Jaffna District. This discrepancy may be due to geographical variations in disease prevalence. Furthermore, kidney disease among elderly individuals in Jaffna was most likely the result of complications of other diseases; thus, the effect of those diseases could mask the individual association of kidney disease with the QOL of elderly individuals in the Jaffna District.

It has been well documented by several previous researchers [14, 16, 17, 32–34] that morbidities are strongly associated with quality of life. Furthermore, in the current study, the overall QOL score was significantly lower among those who had a specific health condition than among those who did not have a specific health condition. Complications associated with chronic diseases may interfere with the standard of living of the elderly population. Further expenditures associated with health conditions can impose financial burdens, which can reduce the QOL of the elderly population. In addition, some disease conditions (psychiatric conditions and hearing and visual impairments) carry social stigma in the traditional society in Jaffna, which could contribute to a lower QOL.

As shown by other studies [14, 17, 35], the overall QOL score of elderly individuals with ADL limitations was significantly lower than that of those without ADL limitations. Limitations in ADL tend to restrict the independence of elderly individuals, which can reduce their sense of wellbeing and, thus, ultimately reduce their quality of life. In the context of Jaffna, the rapid migration and death of younger people during the recent war has led to a greatly increased percentage of elderly individuals and a substantial reduction in the number of caregivers [25], which may further worsen the consequences of ADL limitations; thus, it could have significantly reduced the QOL of elderly individuals.

Satisfaction with one’s health status was found to be an essential contributor to overall quality of life. The elderly individuals who reported being satisfied with their overall health had a higher quality of life score. This finding confirms that health is a vital component of quality of life, as has been indicated by several researchers [14, 16]. In the current study, more than half of the participants reported being satisfied with their health. Furthermore, this positive finding could be associated with the relatively better health service availability in the Jaffna district. Satisfaction with their health may be associated with the physical and psychological wellness of the elderly population. In addition, elderly individuals who feel healthy might be more likely to participate in social activities. These factors might have enhanced their QOL.

Regarding clinical follow-up, elderly individuals who were attending a clinic regularly had a higher quality of life score than the others. Furthermore, the proportion of elderly individuals who were attending a clinic for hypertension and diabetes mellitus was relatively high.

Attending clinical follow-up was associated with a higher QOL in patients with those diseases, even though both diseases were found to be associated with a reduced quality of life in previous studies [36].

It is well known that the need for health services among the elderly population is high compared to other age groups [28]. It was reported that a higher frequency of doctor visits was associated with lower QOL [37]. However, in our study, it was found that elderly persons who visited a doctor at least once per month had significantly higher overall QOL scores than others. This is likely because regular visits to doctors could have enhanced their health and minimised the complications associated with ageing.

Some researchers have argued that the use of addictive substances decreases the quality of life of elderly individuals [38]. However, some other researchers have stated that this factor does not significantly affect the quality of life of elderly individuals [39]. In the current study, addictive substance use was found to negatively affect quality of life.

Jaffna District is known for its tobacco cultivation and cigar industry. One in ten farmers cultivate tobacco, and cigar smoking was is common among elderly individuals in Jaffna [40]. In addition, alcohol consumption is also common in Jaffna district [41], and native Palmyra toddy drinking is common among the elderly population [42]. Betel chewing is seen as a traditional practice, especially among older women in Jaffna. Exposure to addictive substances can lead to adverse health conditions. Furthermore, it can impose costs on elderly individuals. These factors could have contributed to the lower QOL among the elderly individuals who used addictive substances than those who did not.

### Limitations

All the information was self-reported, without any cross-checks of clinical records. Elderly individuals who stated that they had been diagnosed with a disease by a doctor were recorded as having that disease. Therefore, underreporting could be a limitation.

## Conclusions

The quality of life of the elderly population in Jaffna District was found to be average (11.5 ± 2.2 out of 20). As the elderly population is increasing rapidly, policies to improve the QOL of the elderly population are needed. Moreover, it has been found that chronic diseases are becoming increasingly common among elderly individuals in Jaffna District and that musculoskeletal complaints are the major problems in this population. Lifestyle modifications and healthy eating practices should be encouraged to reduce the prevalences of chronic diseases.

It was noted that the quality of life of elderly individuals with diabetes mellitus and hypertension was not as low as that of similar elderly individuals in previous studies. Furthermore, most elderly persons with diabetes mellitus and hypertension attended regular follow-up at clinics and visited a doctor frequently. This showed that these practices had a positive effect on their quality of life. These practices should be encouraged through the provision of additional assistance. It is also notable that the self-reported satisfaction of the participants with their health was higher than the results reported in other similar studies conducted in Sri Lanka.

The prevalence of exposure to addictive substances was found to be higher among the elderly population in Jaffna than the national prevalence, and it was found to be a contributing factor to a reduced QOL. Strict policies to control the use of addictive substances may enhance the QOL of the elderly population. Social values that encourage the use of addictive substances should be eliminated.

The following factors were found to independently contribute to a reduced quality of life among elderly individuals in Jaffna District: (a) the presence of specific health conditions, (b) the presence of musculoskeletal conditions, (c) limitations in activities of daily living, and (d) exposure to addictive substances. On the other hand, satisfaction with their health, visiting a doctor at least once a month, and having diabetes mellitus positively contributed to a better quality of life among the study participants.

Findings from this study can be generalised due to the use of a representative sample. The study had a clear objective, which was to determine the factors associated with QOL; therefore, the contribution of the factors highlighted in the study should be considered when developing interventions to improve the QOL of elderly individuals in the Jaffna district of Sri Lanka.

## Data Availability

The data (questionnaires and datasets) of the current study are available from the corresponding author upon reasonable request.

## Abbreviations

QOL: Quality of life
ADL: Activity of daily living
WHOQOL: World Health Organisation Quality of Life
